# Supplementary material for: Molecular Subtypes and Tumor Microenvironment Characteristics of Small-Cell Lung Cancer Associated with Platinum-Resistance
Source: Cancers (Basel). 2023 Jul 11;15(14):3568. doi: 10.3390/cancers15143568 (PMC10377352; doi:10.3390/cancers15143568)

Figure S1. DEG expression and NE signature score boxplots for each subtype extracted from the George cohort (A) and CCLE (B). P values were acquired by ANOVA.

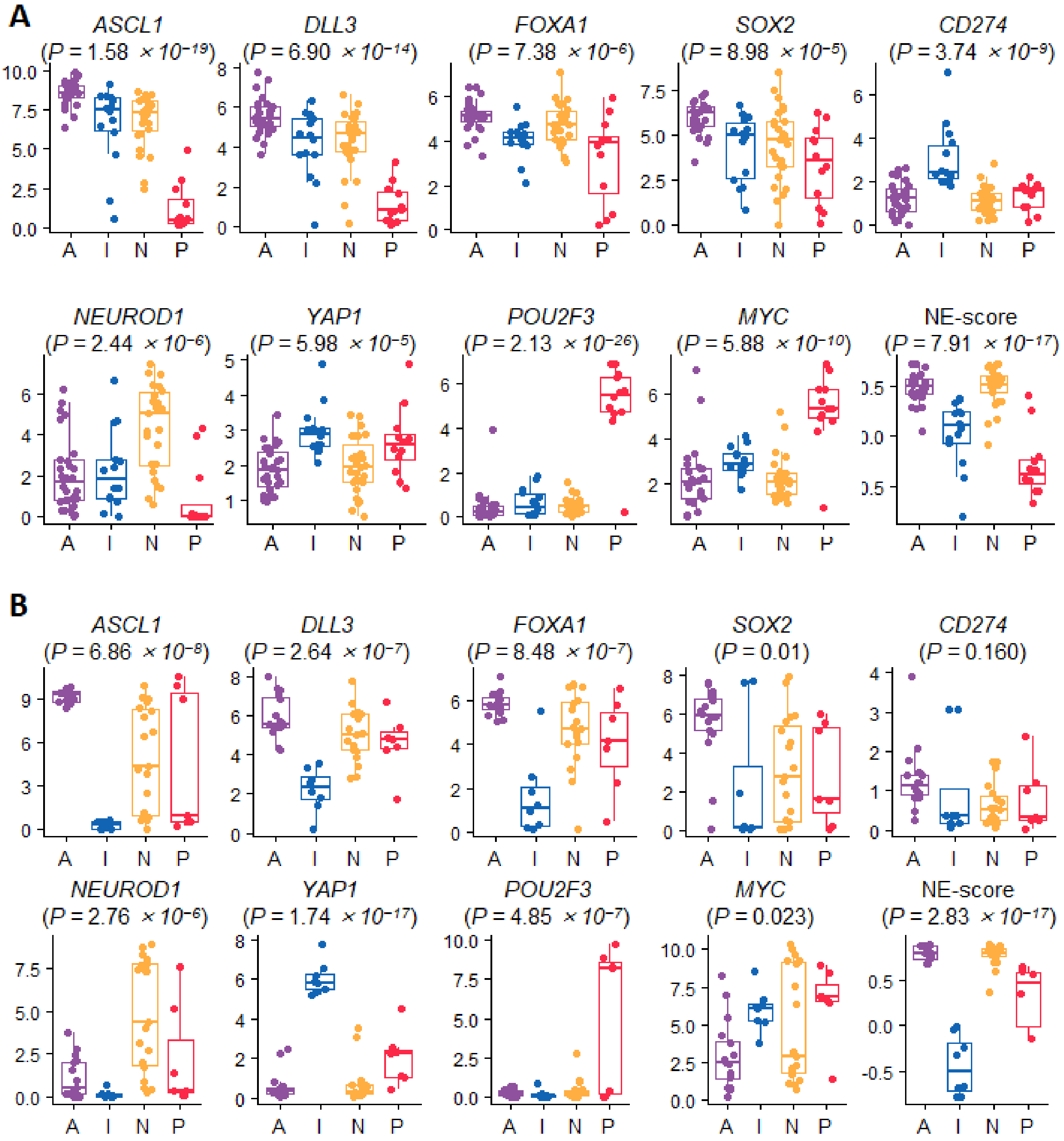

Figure S2. Non-NE cell transcriptome analysis. A) A volcano plot for DEGs to compare NE- and non-NE-type cells. The X-axis is FC, and the Y-axis is -log(*P value*). B) Pathway enrichment analysis of NE and non-NE DEGs. C) t-SNE plots for TME clusters and patients. D) Gene expression status was used for cell type annotation for the five TME subclusters. Circle size indicates the fraction of gene-expressing cells, and the color scale indicates the average expression. E) Boxplots of POU2F3 transcription factor target gene expression to identify the SCLC-P type.

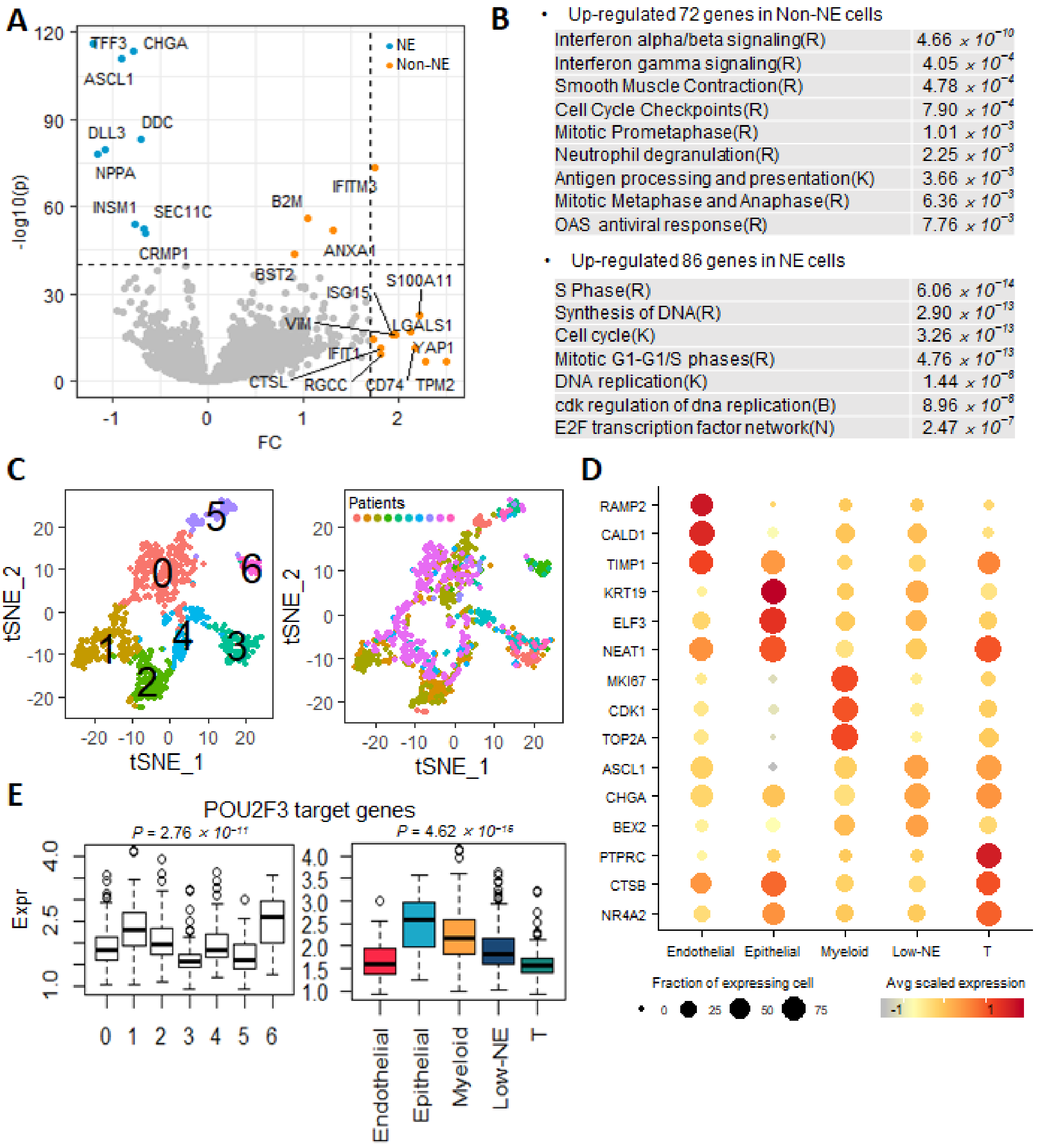

Figure S3. IHC results of four samples for each SCLC subtype. A) SCLC-A, B) SCLC-I, C) SCLC-N, and D) SCLC-P. The status of CD4, CD8, CD20, FOXP3, PD-L1 and CK was investigated on patient slides.

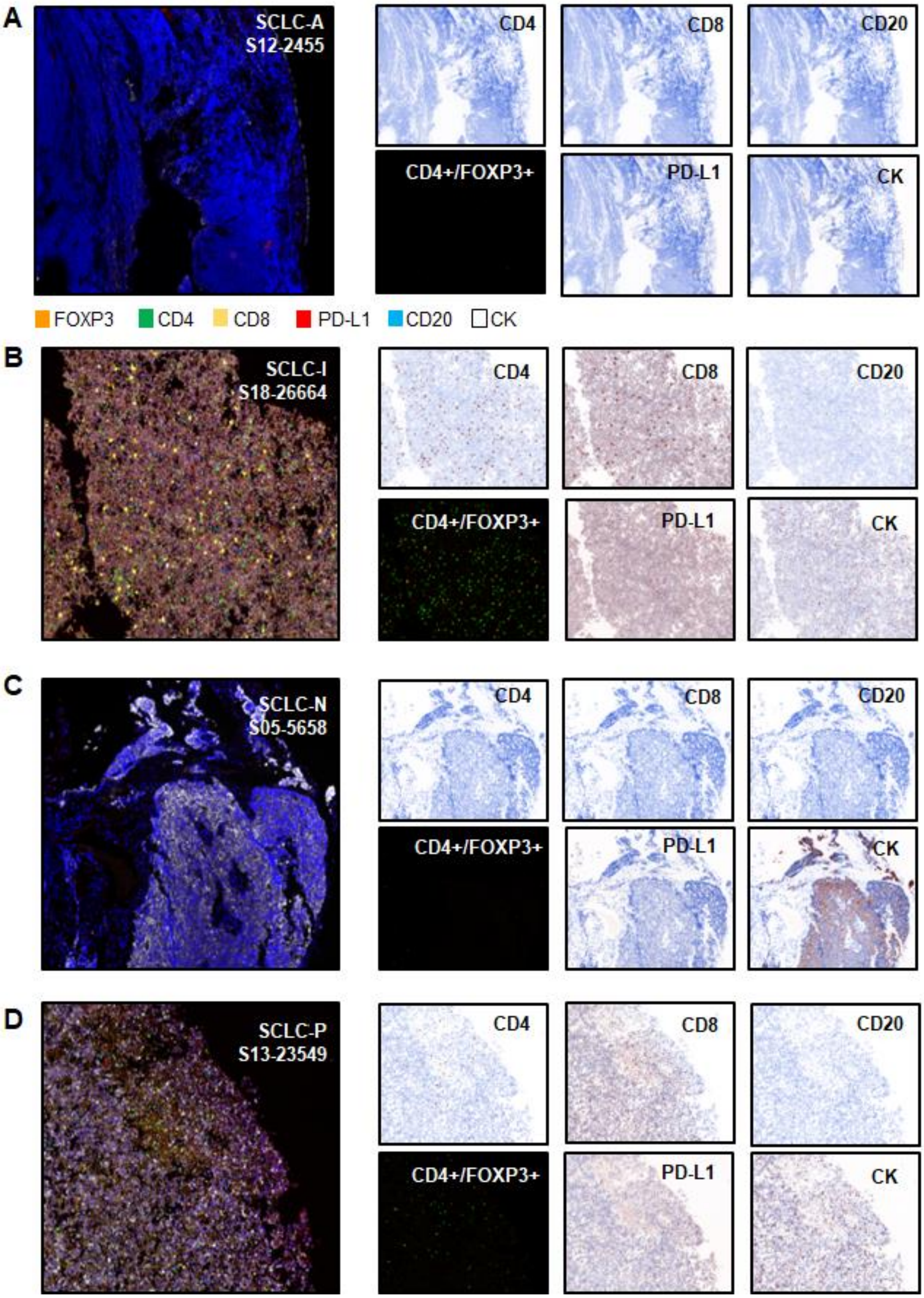

Figure S4. Analysis of two-year PFS for immune cell types in the NCC cohort.

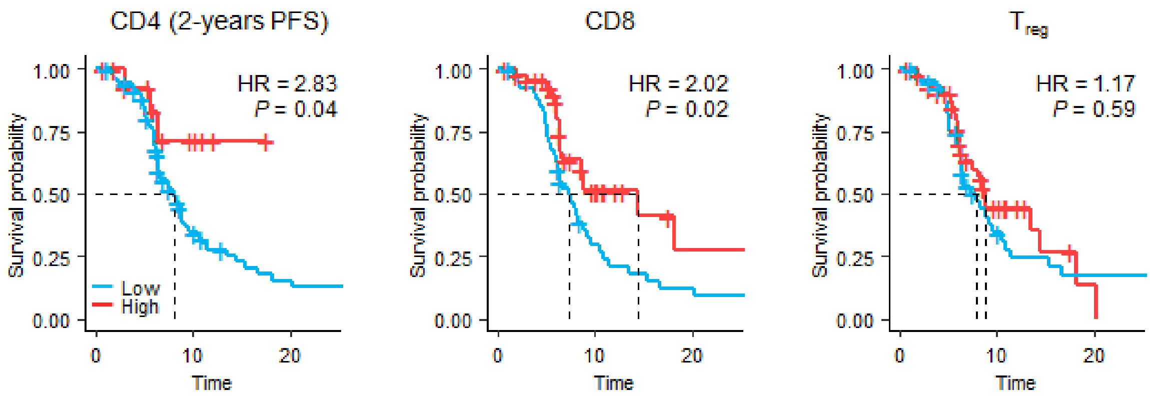

Supplement: Supplementary file 1 [file cancers-15-03568-s001.zip › supplementary_fig.pdf]
